# Myeloperoxidase-antineutrophil cytoplasmic antibody-associated crescentic glomerulonephritis in autosomal dominant polycystic kidney disease

**DOI:** 10.1186/1471-2369-14-94

**Published:** 2013-04-23

**Authors:** Keiichi Sumida, Yoshifumi Ubara, Junichi Hoshino, Noriko Hayami, Tatsuya Suwabe, Rikako Hiramatsu, Eiko Hasegawa, Masayuki Yamanouchi, Naoki Sawa, Kenmei Takaichi, Kenichi Ohashi

**Affiliations:** 1 Nephrology Center, Toranomon Hospital, Tokyo, Japan; 2 Department of Pathology, Toranomon Hospital, Tokyo, Japan

**Keywords:** Myeloperoxidase, Antineutrophil cytoplasmic antibody, Crescentic glomerulonephritis, Autosomal dominant polycystic kidney disease

## Abstract

**Background:**

Autosomal dominant polycystic kidney disease (ADPKD) is an inherited disorder that is characterized by the development of cysts in the kidneys and other organs. Urinary protein excretion is usually less than 1 g/day, and ADPKD is rarely associated with nephrotic syndrome or rapidly progressive glomerulonephritis (RPGN). To date, myeloperoxidase (MPO)-antineutrophil cytoplasmic antibody (ANCA)-associated crescentic glomerulonephritis (CrGN) has not been reported in a patient with ADPKD.

**Case presentations:**

We report two cases of MPO-ANCA positive ADPKD. A 60-year-old Japanese woman (case 1) and a 54-year-old Japanese woman (case 2) presented with RPGN featuring severe proteinuria and microscopic hematuria. In both patients percutaneous needle biopsy of the kidney revealed MPO-ANCA-associated CrGN with a paucity of glomerular immunoglobulin staining. Each patient received intravenous methylprednisolone for 3 days, followed by oral prednisolone. Case 1 showed gradual improvement and has not progressed to end-stage renal disease (ESRD), but case 2 developed ESRD requiring hemodialysis within one month despite treatment.

**Conclusion:**

These are the first two reported cases of MPO-ANCA-associated CrGN in patients with ADPKD. Our experience suggests that serial measurement of the ANCA titer and renal biopsy should be considered for accurate diagnosis and appropriate treatment of ADPKD patients who present with proteinuria, hematuria, and rapid decline of renal function.

## Background

Autosomal dominant polycystic kidney disease (ADPKD) is a common inherited disorder that is characterized by the development of cysts in the kidneys and other organs, with its prevalence estimated to be between 1 in 400 and 1 in 1000 individuals. In most patients, urinary protein excretion is less than 1 g/day and renal function is maintained within the normal range despite the relentless growth of cysts, until they reach the fourth to sixth decade of life. Then renal function starts to decline, with the glomerular filtration rate decreasing by approximately 4.4–5.9 ml/min/year [1,2]. Therefore, detection of nephrotic range proteinuria, hematuria, and/or a rapid decline of renal function in ADPKD patients should suggest the possibility of complicating glomerular disease [3]. Although various histopathological lesions have been reported in ADPKD patients, including focal segmental glomerulosclerosis (FSGS), membranous nephropathy, minimal change disease, and immunoglobulin A (IgA) nephropathy [4-18], myeloperoxidase (MPO)-antineutrophil cytoplasmic antibody (ANCA)-associated crescentic glomerulonephritis (CrGN) has not been reported so far.

Here we describe the first two cases of MPO-ANCA-associated CrGN in ADPKD patients who presented with rapidly progressive glomerulonephritis (RPGN), severe proteinuria, and microscopic hematuria.

## Case presentations

### Case 1

In September 2010, a 60-year-old Japanese woman was admitted to a general hospital with the symptom of abdominal pain and nausea. ADPKD had been diagnosed in 2000 when she had a slightly elevated serum creatinine level (1.3 mg/dL) without proteinuria or hematuria. Her medical history included hypertension that was being treated with an antihypertensive agent (losartan potassium 50 mg/day).

**Figure 1:**
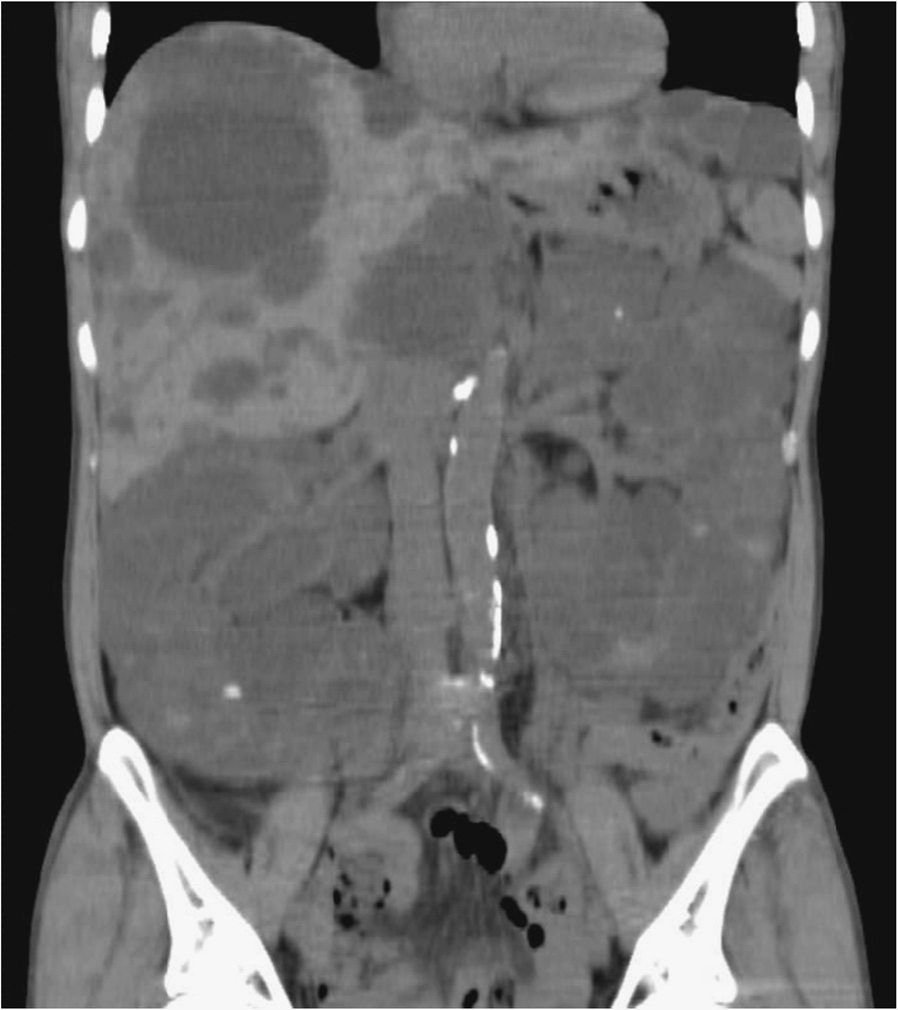
Abdominal computed tomography shows an enlarged liver and kidneys with multiple cysts in case 1.

On admission, her temperature was 36.2°C and her blood pressure was 117/70 mmHg. Physical examination was normal, except for bilateral palpable lumpy kidneys. Hematology tests indicated a white blood cell count of 4.6 × 10^9^/L, hemoglobin of 9.0 g/dL, and platelet count of 24.3 × 10^9^/L. Blood urea nitrogen was 46 mg/dL, serum creatinine was 4.5 mg/dL, serum albumin was 3.7 g/dL, C-reactive protein (CRP) was 0.96 mg/dL, rheumatoid factor was 16 U/mL, antinuclear antibody was 1:40 (normal range: <1:80), and MPO-ANCA was 393 EU (normal range: <20 EU). Serum IgG, IgA, IgM, total hemolytic complement (CH50), and complement components were normal. Cryoglobulinemia, anti-glomerular basement membrane (GBM) antibody, and anti-proteinase 3 (PR3) antibody were negative. Urinalysis showed proteinuria (2.03 g/day). The sediment contained over 100 dysmorphic red blood cells (RBCs) per high power field (HPF) with RBC casts and 1 to 5 white blood cells (WBCs)/HPF. Creatinine clearance (C_Cr_) was 10.2 ml/min. Blood and urine cultures were negative. The chest X-ray film and computed tomography (CT) scan were normal. Abdominal ultrasonography (US) and CT showed enlargement of the liver and kidneys with multiple cysts (Figure 1).

MPO-ANCA-associated RPGN was suspected and the patient was treated with prednisolone (PSL) at 35 mg/day. Within one month, her serum creatinine declined to 3.2 mg/dL, but proteinuria and hematuria persisted at 3.50 g/day and 10 to 30 RBCs/HPF, respectively. She was referred to our hospital for further examination and treatment. To make a definite diagnosis, percutaneous needle biopsy of the kidney was performed in October 2010.

**Figure 2:**
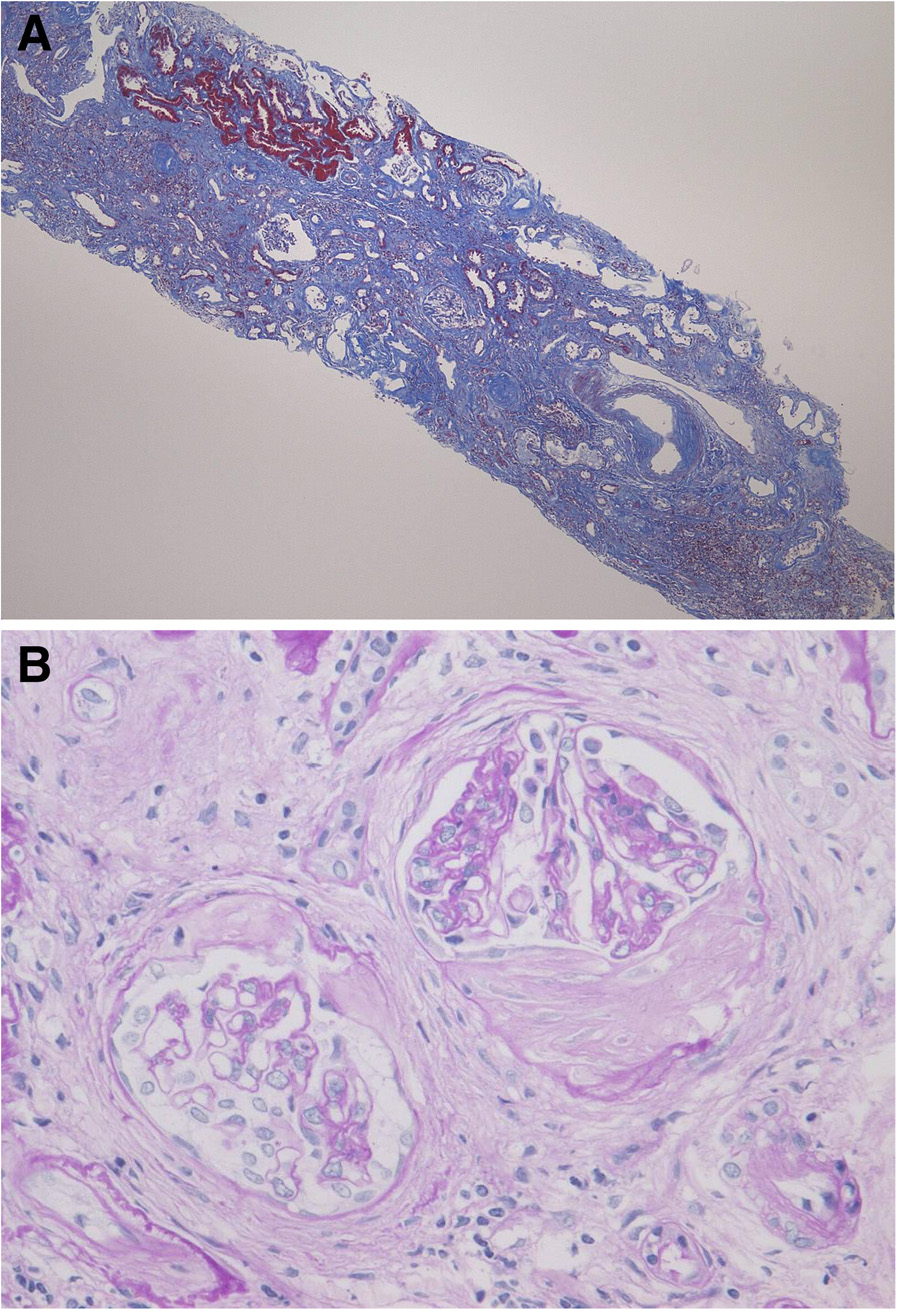
**Light microscopy findings on renal biopsy in case 1.** (**A**) Severe inflammatory interstitial fibrosis and tubular atrophy with mild hyaline arteriolosclerosis and moderate intimal thickening of the interlobular artery. (Masson-trichrome stain; original magnification × 40). (**B**) The glomeruli show fibrous crescent formations and disruption of Bowman’s capsule with periglomerular fibrosis. (Periodic acid-Schiff stain; original magnification × 400).

On light microscopy, 19 glomeruli were observed, with 6 being sclerotic. Of the 13 nonsclerotic glomeruli, 3 showed fibrous crescent formation and the others were relatively preserved with segmental capillary wall wrinkling. Severe inflammatory interstitial fibrosis and tubular atrophy were also observed, along with mild hyaline arteriolosclerosis and moderate intimal thickening of the interlobular artery (Figure 2A and B). There was no vasculitis of the arteries or arterioles. Immunofluorescence microscopy showed negative staining of the glomeruli. Electron microscopy showed no immune complex deposits. It was considered that these findings reflected the status after treatment of MPO-ANCA-associated CrGN with PSL. She was given intravenous methylprednisolone (500 mg/day) for 3 days, followed by oral PSL (20 mg/day), resulting in further improvement of her renal function as well as the proteinuria and hematuria. In July 2012 (21 months after the onset), her serum creatinine was 2.8 mg/dL, the MPO-ANCA titer was 25 EU, and proteinuria was 0.2 g/day without hematuria. She is being maintained on 5 mg/day of oral PSL.

### Case 2

The patient was a 54-year-old Japanese woman, in whom ADPKD had been diagnosed in 2002 when she had a slightly elevated serum creatinine level (1.3 mg/dL) without proteinuria or hematuria. In September 2010, serum creatinine was increased to 1.9 mg/dL. It rose further to 5.7 mg/dL with proteinuria (2.9 g/gCr) and hematuria by November 2010, and a high MPO-ANCA titer of 210 EU (normal range: <20 EU) was also detected at that time. She was admitted to our hospital for further evaluation. Her medical history included subarachnoid hemorrhage at the age of 36 years.

**Figure 3:**
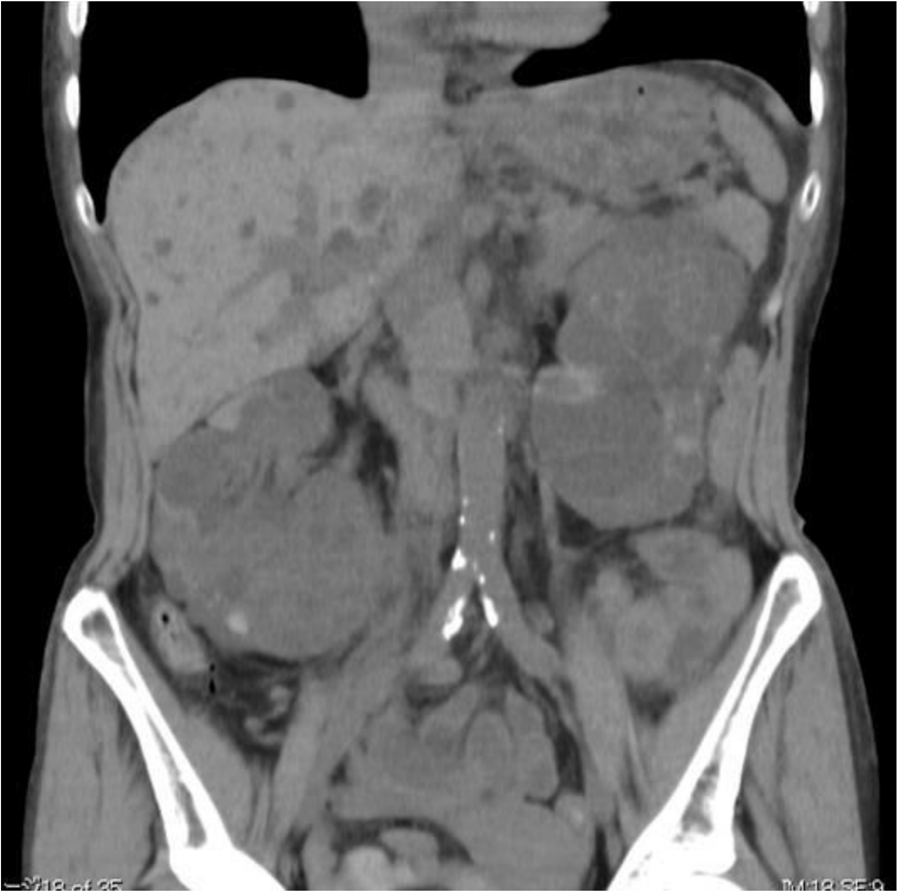
Abdominal computed tomography shows an enlarged liver and kidneys with multiple cysts in case 2.

On admission, her temperature was 36.8°C and her blood pressure was 148/80 mmHg. Physical examination was normal, except for bilateral palpable lumpy kidneys. Hematology tests revealed a white blood cell count of 6.5 × 10^9^/L, hemoglobin of 7.9 g/dL, and platelet count of 20.4 × 10^9^/L. Blood urea nitrogen was 83 mg/dL, serum creatinine was 5.7 mg/dL, serum albumin was 3.0 g/dL, CRP was 1.1 mg/dL, antinuclear antibody was 19.6 index (normal range: <20 index), and MPO-ANCA was 210 EU. Serum IgG, IgA, IgM, CH50, and components of complement were normal. Investigations for cryoglobulinemia, anti-GBM antibody, and anti-PR3 antibody were negative. Urinalysis showed proteinuria (3.85 g/day) and the sediment contained over 100 dysmorphic RBCs/HPF along with RBC casts and 1 to 5 WBCs/HPF. C_Cr_ was 15.2 ml/min. Blood and urine cultures were negative. The chest X-ray film and CT were normal, while abdominal US and CT showed enlargement of the liver and kidneys with multiple cysts (Figure 3).

**Figure 4:**
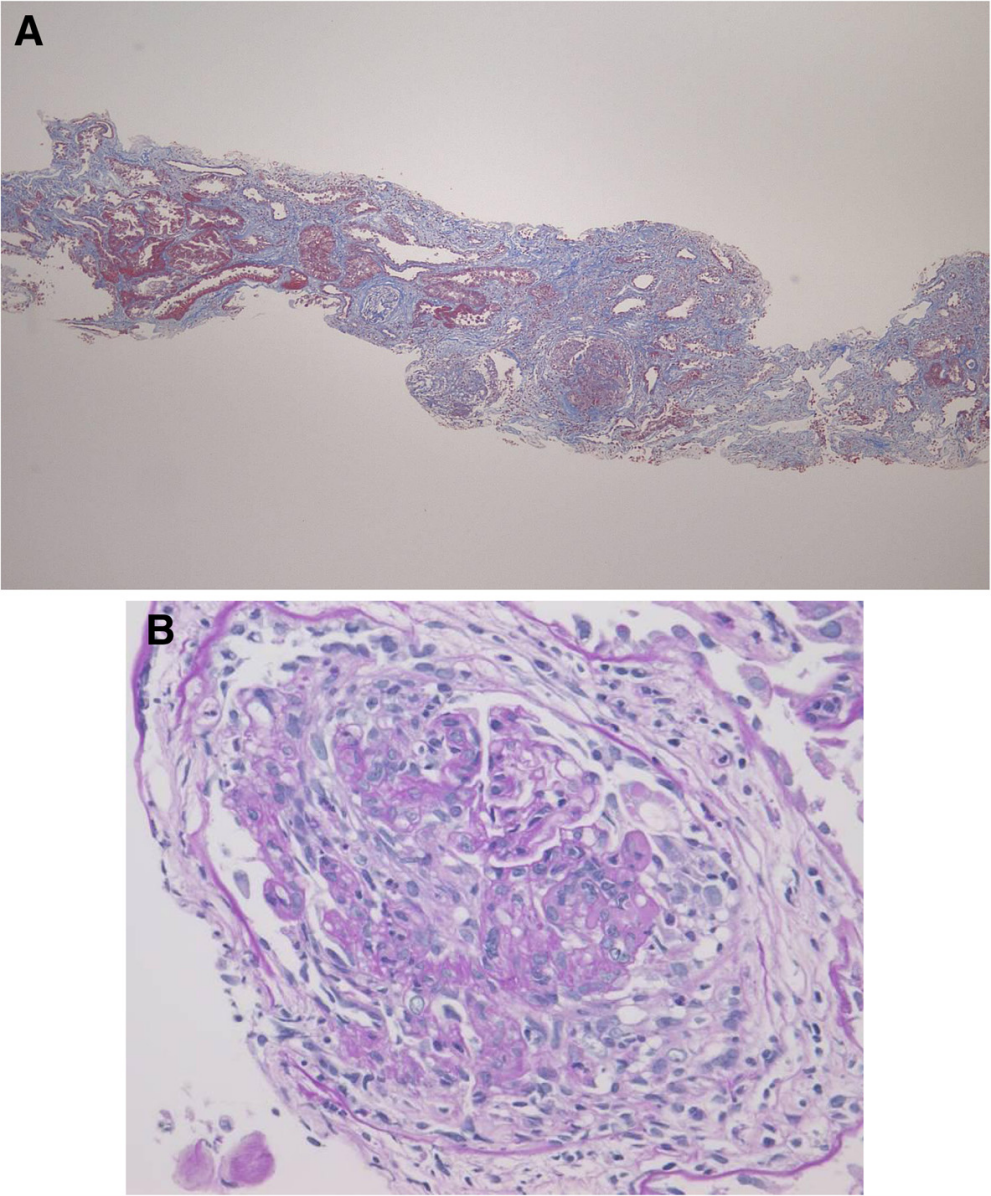
**Light microscopy findings on renal biopsy in case 2.** (**A**) Moderate inflammatory interstitial fibrosis and tubular atrophy, as well as focal cystic and papillary changes of the tubules that are characteristic of polycystic kidney disease. (Masson-trichrome stain; original magnification × 40). (**B**) The glomeruli show cellular crescent formation and partial disruption of Bowman’s capsule. (Periodic acid-Schiff stain; original magnification × 400).

Percutaneous renal biopsy was performed. On light microscopy, 9 glomeruli were observed, none of which were sclerotic. Six of the 9 glomeruli showed cellular crescent formation. Moderate inflammatory interstitial fibrosis and tubular atrophy were observed, as well as focal cystic and papillary changes of the tubules that are characteristic of polycystic kidney disease (Figure 4A and B). There was no evidence of vasculitis affecting the arteries or arterioles. Immunofluorescence microscopy showed negative staining of the glomeruli and electron microscopy detected no immune complex deposits. These results supported a diagnosis of MPO-ANCA-associated CrGN with ADPKD. Intravenous methylprednisolone (500 mg/day) was given for 3 days immediately after the renal biopsy, followed by 30 mg/day of oral PSL. However, her renal function continued to deteriorate. Even three courses of plasma exchange failed to suppress disease activity and exacerbation of renal dysfunction led to end-stage renal disease (ESRD) requiring hemodialysis within one month of admission. In July 2012 (20 months after the onset), she was on maintenance hemodialysis and was receiving oral PSL (2 mg/day) with no recurrence of vasculitis. At that time, CRP was 0.3 mg/dL and the MPO-ANCA titer was less than 10 EU.

### Discussion

The most common causes of a sudden decline of renal function in ADPKD patients are volume depletion, obstruction, and infection [19]. Hematuria (which is usually gross) occurs at some time during the course in 35 to 50% of patients with ADPKD and may even be the presenting symptom of this disease [20,21]. In contrast to macroscopic hematuria, microscopic hematuria (especially with RBC casts) is rarely seen in ADPKD patients. Therefore, the detection of proteinuria in the nephrotic range, hematuria with RBC casts, and/or a rapid decline of renal function in ADPKD patients suggests the possibility of superimposed glomerular diseases.

Seventeen cases of ADPKD associated with glomerular disease that was evaluated by renal biopsy have been reported in the English literature to date (Table 1). Among these 17 patients, 4 had FSGS and this represented the most common histological subtype associated with ADPKD [3-6], followed by 3 cases of membranous nephropathy [7-9], 2 cases each of minimal change disease [10,11], IgA nephropathy [12,13], and post-infectious mesangial proliferative glomerulonephritis [14,15], and one case each of membranoproliferative glomerulonephritis [15], mesangioproliferative glomerulonephritis [16], diabetic glomerulosclerosis [17], and crescentic glomerulonephritis [18]. The high incidence of FSGS suggests that glomerular hyperfiltration could play an important role in the development of FSGS and heavy proteinuria in ADPKD patients, while FSGS may in turn be important for progression to ESRD in a subgroup of ADPKD patients [3]. However, it is difficult to be certain whether these glomerular diseases are coincidental or whether they demonstrate a specific pathogenetic relationship with ADPKD.

**Table 1:** List of patients who had ADPKD and glomerular disease with biopsy-proven renal histopathology

**Patient no.**	**First author (Ref. No.)**	**Age**	**Gender**	**Serum albumin (g/dL)**	**Cr (mg/dL)**	**Proteinuria (g/day)**	**Hematuria (/HPF)**	**Renal biopsy**	**Renal histopathology**	**Treatment**	**Follow-up period**	**Outcome**
1	Murphy [4]	44	M	3.4	9.9	7	NA	O	FSGS	ACEi	32 months	HD
2	Montoyo [5]	35	M	2.4	3.5	14	NA	O	FSGS	ACEi	3 months	HD
3	Dionisio [6]	58	M	NA	Slight increase	8	NA	O	FSGS	Steroid, ACEi	6 years	HD
4	Contreas [3]	65	F	3.7	1.2	5.8	NA	O	FSGS	ACEi	3 years	C_Cr_ 32 ml/min, proteinuria 5.5 g/day
5	Shikata [7]	53	F	2.2	1.0	6	3-5	O	MN	Pred,	NA	NA
6	Saxena [8]	22	M	2.5	0.8	6	occasional	NA	MN	NA	NA	NA
7	Peces [9]	38	M	NA	1	11.8	NA	P	MN	ARB, Pred, MMF	10 years	C_Cr_ 114 ml/min, proteinuria 0.4 g/day
8	Nakahama [10]	14	M	1.7	C_Cr_ 114 ml/min	23.0	A few	P	MCD	MP, Pred	1 months	Remission
9	Kuroki [11]	18	F	3.4	0.9	5.4	0	O	MCD	Pred, CPA	6 months	Remission
10	Panisello [12]	67	M	4.4	6.3	4.2	10	O	IgAN	NA	3 months	Cr 9.2 mg/dL
11	Hiura [13]	70	M	2.2	1.69	5.76	>100	O	IgAN	Pred	5 years	C_Cr_ 40.9 ml/min, proteinuria 0.08 g/day
12	D’Cruz [14]	35	M	3.0	1.7	4.67	8-10	O	PIGN	ARB	12 weeks	Remission
13	Villar [15]	28	M	NA	5.7	4.7	NA	O	PIGN	ACEi	1 year	PD
14	Villar [15]	25	M	2.7	5.5	12	NA	O	MPGN	MP	10 months	PD
15	Seyrek [16]	56	F	NA	NA	0.5	many	NA	MesGN	Pred	1 year	Remission
16	Hariharan [17]	44	M	2.5	1.35	11	10-12	P	IDGS	NA	1 year	HD
17	Licina [18]	69	F	NA	5	(3+)	Many	O	CrGN	MP, Pred	3 months	Cr 2.4 mg/dL
18	Present case 1	60	F	3.7	4.5	2.03	>100	P	ANCA-associated CrGN	MP, Pred	21 months	Cr 2.8 mg/dL, proteinuria 0.2 g/day
19	Present case 2	54	F	3.0	5.7	3.85	>100	P	ANCA-associated CrGN	MP, Pred, PEX	1 month	HD

NA, not available; P, percutaneous biopsy; O, open surgical biopsy; FSGS, focal segmental glomerulosclerosis; MN, membranous nephropathy; MCD, minimal change disease; IgAN, IgA nephropathy; PIGN, post-infectious mesangial proliferative glomerulonephritis; MPGN, membranoproliferative glomerulonephritis; MesGN, mesangioproliferative glomerulonephritis; IDGN, intercapillary diabetic glomerulosclerosis; CrGN, crescentic glomerulonephritis; ANCA, antineutrophil cytoplasmic antibody; ACEi, angiotensin-converting enzyme inhibitor; ARB, angiotensin II receptor blocker; Pred, prednisone; MMF, mycophenolate mofetil; MP, methylprednisolone; PEX, plasma exchange; Cr, serum creatinine; C_Cr_, creatinine clearance; HPF, high power field; HD, hemodialysis; PD, peritoneal dialysis.

There has only been one report of ADPKD associated with RPGN due to CrGN, which was a case described by Licina *et al.* in 1981 [18]. Because the patient had acute renal failure and microscopic hematuria with RBC casts, they performed open renal biopsy and this led to the diagnosis of idiopathic crescentic RPGN. The actual diagnosis could have been ANCA-associated CrGN, but this case occurred before the discovery of ANCA by Davies *et al.* in 1982 [21]. In Asian countries, the prevalence of MPO/PR3-ANCA in patients with ANCA-associated vasculitis (AAV) is much higher than in European countries [22,23], but there has been no report about MPO-ANCA-associated vasculitis in ADPKD patients or any association between MPO-ANCA and ADPKD. Regarding the pathogenesis of AAV, recent studies have indicated a triggering role of microbial factors. In particular, *Staphylococcus aureus* carrier status and infection with Gram-negative bacteria could contribute to the onset and persistence of AAV [24,25]. Kain et al. identified autoantibodies to human lysosome-associated membrane protein-2 (hLAMP-2) in patients with pauci-immune necrotizing glomerulonephritis (NCGN) who were positive for PR3-ANCA or MPO-ANCA. They proposed that such autoantibodies might contribute to renal injury because the antigen is expressed on the plasma membrane of glomerular endothelial cells. They also revealed that an immunodominant epitope of hLAMP-2 showed strong homology with FimH, an adhesion protein of Gram-negative bacteria such as *Escherichia coli* and *Klebsiella pneumonia*, and suggested that Gram-negative infection might induce pathogenic autoantibodies in a susceptible host, resulting in NCGN [25]. Although bacterial infection was not detected in our two patients, a subclinical Gram-negative infection (such as a latent cyst infection) could possibly have contributed to the pathogenesis of MPO-ANCA-associated CrGN.

In ADPKD patients, the presence of multiple cysts in both kidneys is considered as a contraindication to percutaneous renal biopsy due to the presumed risk of complications and difficulty in obtaining suitable tissue for diagnosis. Indeed, only 3 of the 17 patients (17.6%) listed in Table 1 underwent percutaneous renal biopsy, while 13 patients (76.5%) had open surgical biopsy. In the remaining one patient, the details of the procedure were unknown. In our two cases, abdominal computed tomography was initially performed to confirm the site of residual renal parenchyma, after which percutaneous needle biopsy was performed without complications. This enabled us to make a definite diagnosis of MPO-ANCA-associated CrGN and to provide appropriate corticosteroid therapy with confidence. Although our experience with percutaneous needle renal biopsy is too limited to recommend its widespread adoption, US-guided needle biopsy is less invasive and fewer complications, so it is worth considering when renal biopsy is required in ADPKD patients.

## Conclusion

To the best of our knowledge, this is the first report about MPO-ANCA-associated CrGN in ADPKD patients. These two cases emphasize that detection of proteinuria, hematuria (especially with RBC casts), and a rapid decline of renal function in ADPKD patients should suggest the possibility of glomerular disease. Then serial measurement of ANCA and renal biopsy should be considered to allow accurate diagnosis and appropriate treatment.

## Consent

Written informed consent was obtained from both patients for publication of their case reports and any accompanying images. A copy of the written consent is available for review by the Editor of this journal.

## Competing interests

The authors declare that they have no competing interests.

## Authors’ contributions

KS, YU, JH, NH, TS, RH, EH, MY, NS and KT treated the patients and provided data about the history and laboratory results. KO interpreted the renal biopsies. KS drafted the manuscript. All authors read and approved the final manuscript.

## Pre-publication history

The pre-publication history for this paper can be accessed here:

http://www.biomedcentral.com/1471-2369/14/94/prepub
